# Comment on: Validation of the Southend giant cell arteritis probability score in a Scottish single-centre fast-track pathway. Reply

**DOI:** 10.1093/rap/rkac042

**Published:** 2022-05-19

**Authors:** Andrew R Melville, Karen Donaldson, James Dale, Anna Ciechomska

**Affiliations:** Institute of Infection, Immunity & Inflammation, University of Glasgow, Glasgow; Rheumatology Department, University Hospital Wishaw, NHS Lanarkshire, Wishaw, UK; Rheumatology Department, University Hospital Wishaw, NHS Lanarkshire, Wishaw, UK; Rheumatology Department, University Hospital Wishaw, NHS Lanarkshire, Wishaw, UK

Dear Editor, We thank Marieke van Nieuwland *et al.* [1] for their comments on our recent article [2] and for sharing their experience of using the Southend GCA probability score (GCAPS) in their centre in The Netherlands. Interestingly, and in contrast to our findings and those of other UK authors [3, 4], several of their GCA patients had GCAPS < 9 (5/40, or 12.5%).

Two important factors that might explain our discrepant results (aside from genuine phenotypic differences between GCA cohorts) are the approaches to GCAPS scoring and the confirmation of GCA diagnoses.

In our study, GCAPS components were documented methodically and contemporaneously by a consultant rheumatologist during clinical assessment. Our diagnostic process was robust, in that all patients underwent US evaluation, US findings were interpreted according to clearly defined criteria, and temporal artery biopsies (TABs) were arranged if needed (e.g. if US was inconclusive). Most diagnoses (>90%) were supported by positive US or TAB, and cases were confirmed clinically at 6 months. Since publication, we have continued data collection [5]; as of March 2022, 208 patients have been assessed on our fast-track pathway, with 64 diagnosed with GCA (30.8%). All new GCA diagnoses made between December 2020 and March 2022 have been supported by positive US or TAB. Still, none of our GCA patients has had GCAPS < 10.

We note that in the study by van Nieuwland *et al.* [6], GCAPS scoring appears to have been applied retrospectively from review of case notes, and in 74 of 209 subjects there were insufficient data for inclusion. It is conceivable, therefore, that GCAPS components were missed in some cases, contributing to relatively low scores. Indeed, extracranial vascular signs were excluded entirely (hence the term modified GCAPS), albeit these are relatively rare signs detected in only ∼4% of our cohort. There are also notable differences when comparing characteristics of patients diagnosed with GCA between studies. In the study by van Nieuwland *et al.* [6], more relatively young patients were diagnosed (including one <50 years *vs* none in our study, and ∼12% <60 years *vs* ∼7% in our study), and several patients had limited evidence of a systemic inflammatory response (three patients had CRP  < 5 mg/L and two patients had CRP < 10 mg/L, *vs* none with CRP < 10 mg/L in our study). Although the diagnostic approaches appear similar between studies, the relative use of US, TAB and fluorodeoxyglucose (FDG)-PET or CT is unclear in the study by van Nieuwland *et al.* [6], and the criteria used for US interpretation are not given, both of which would affect overall confirmatory test specificity and, perhaps, positive diagnosis rates. Furthermore, routes to diagnosis for low-GCAPS patients specifically are not detailed, precluding a better understanding of the phenotype of this unusual subgroup.

Van Nieuwland *et al.* [1] speculate that we might have overlooked atypical presentations of GCA with low GCAPS scores, in part because we rejected ∼30% of fast-track referrals on grounds of clinical implausibility. We feel this is unlikely. There is only one referral pathway for GCA in our clinical catchment area (NHS Lanarkshire), and we have not been made aware of any missed cases. Furthermore, as part of ongoing service evaluation we have collected additional data between April 2021 and March 2022. Of 111 referrals, 41 were rejected before formal assessment (36.9%); only 1 of 41 was subsequently re-referred, with GCA excluded after clinical and US assessment.

There is now growing real-world evidence for the effectiveness of GCAPS as a risk-stratification tool. However, the differences in clinical practice and/or patient populations highlighted here suggest that the role GCAPS could play in the fast-track pathway of a specific centre will vary according to local factors, including service availability or restrictions.

In NHS Lanarkshire, GCAPS plays a central role in triage and has now been incorporated into the referral pathway from primary care. As safeguards, we have implemented enhanced vetting of referrals (with review of clinical notes and test results, requests for additional information from the referrer, or telephone consultations, as necessary), in addition to ongoing service evaluation as described above. We feel that this helps to protect the service from inappropriate referrals, thus reducing waiting times for patients truly at risk, enhancing the performance of additional tests, improving diagnostic accuracy and ensuring appropriate treatment.

*Funding:* No specific funding was received from any bodies in the public, commercial or not-for-profit sectors to carry out the work described in this article.

*Disclosure statement:* A.R.M. is a Medical Research Council (MRC) and GlaxoSmithKline (GSK) Experimental Medicine Initiative to Explore New Therapies clinical training fellow, with project funding outside the submitted work. K.D. has received speaker fees from Menarini Pharma UK. A.C. has received speaker, travel and registration fee sponsorship from Novartis, Abbvie, Chugai Pharma, Colgene, Roche and Lilly. J.D. has declared no conflicts of interest.

## Data availability statement

Data underlying this article will be shared on reasonable request to the corresponding author.
